# Adipose-derived mesenchymal stem cells employed exosomes to attenuate AKI-CKD transition through tubular epithelial cell dependent Sox9 activation

**DOI:** 10.18632/oncotarget.19979

**Published:** 2017-08-07

**Authors:** Fengming Zhu, Octavia L.S. Chong Lee Shin, Guangchang Pei, Zhizhi Hu, Juan Yang, Han Zhu, Meng Wang, Jingyi Mou, Jie Sun, Yuxi Wang, Qian Yang, Zhi Zhao, Huzi Xu, Hui Gao, Weiqi Yao, Xiao Luo, Wenhui Liao, Gang Xu, Rui Zeng, Ying Yao

**Affiliations:** ^1^ Division of Nephrology, Tongji Hospital, Tongji Medical College, Huazhong University of Science and Technology, Wuhan 430030, Hubei, China; ^2^ Department of Clinical Nutrition, Tongji Hospital, Tongji Medical College, Huazhong University of Science and Technology, Wuhan 430030, Hubei, China; ^3^ Wuhan Institute of Biotechnology, Guanggu Biolake, Wuhan 430000, Hubei, China; ^4^ Department of Plastic Surgery, Tongji Hospital, Tongji Medical College, Huazhong University of Science and Technology, Wuhan 430030, Hubei, China; ^5^ Department of Geriatrics, Tongji Hospital, Tongji Medical College, Huazhong University of Science and Technology, Wuhan 430030, Hubei, China

**Keywords:** adipose-derived mesenchymal stem cells, exosomes, Sox9, AKI-CKD, tubular epithelial cells

## Abstract

Acute kidney injury (AKI) predisposes patients to an increased risk into progressive chronic kidney disease (CKD), however effective treatments are still elusive. This study aimed to investigate the therapeutic efficacy of human adipose-derived MSCs (hAD-MSCs) in the prevention of AKI-CKD transition, and illuminate the role of Sox9, a vital transcription factor in the development of kidney, in this process. C57BL/6 mice were subjected to unilateral renal ischemia/reperfusion (I/R) with or without hAD-MSC treatment. We found that hAD-MSC treatment upregulated the expression of tubular Sox9, promoted tubular regeneration, attenuated AKI, and mitigated subsequent renal fibrosis. However, these beneficial effects were abolished by a drug inhibiting the release of exosomes from hAD-MSCs. Similarly, Sox9 inhibitors reversed these protective effects. Further, we verified that hAD-MSCs activated tubular Sox9 and prevented TGF-β1-induced transformation of TECs into pro-fibrotic phenotype through exosome shuttling *in vitro*, but the cells did not inhibit TGF-β1-induced transition of fibroblasts into myofibroblasts. Inhibiting the release of exosomes from hAD-MSCs or the expression of Sox9 in TECs reversed these antifibrotic effects. In conclusion, hAD-MSCs employed exosomes to mitigate AKI-CKD transition through tubular epithelial cell dependent activation of Sox9.

## INTRODUCTION

AKI is an increasingly clinical problem associated with high morbidity and mortality, especially among intensive care unit patients (>50%) [1-3]. Despite the fact that some AKI patients returned to normal renal function after supportive treatments, it shows a 25 percent increase in the risk of dormant progression to CKD and a 50 percent increase in mortality over a ten year follow-up [4, 5]. Recent epidemiological study strongly supports the hypothesis that AKI and CKD are intrinsically tied syndromes [6]. The pathophysiology of AKI is characterized as renal tubular epithelial cell damage, inflammatory infiltration and vascular dysfunction, which initiates the self-repair mechanism of the kidney [7, 8]. Nevertheless, the regenerative capacity of kidney is relatively limited compared to heart, liver and other powerful organs [9]. Generally, the injured kidney generates an inflammatory milieu, which inhibits the migration, homing and paracrine capacity of endogenous stem and progenitor cells into kidney [10]. Thus, maladaptive repair presents as the persistent proliferation, activation of myofibroblasts and excellular matrix deposited in the renal interstitium [11, 12], which are the hallmarks of AKI to CKD transition. However the underlying mechanisms of AKI to CKD progression are still incompletely understood, making the search for new and efficient therapheutic strategies an urgent necessity.

MSCs are adult stem cells with abilities of self-renewal and multi-potent differentiation into mesodermal lineage cells [13], which are used to attenuate AKI induced by cisplatin [14, 15], hypertonic glycerol [16] and folic acid [17]. However, the mechanisms remain controversial. Fate tracing studies demonstrated that exogenous transplanted MSCs failed to directly differentiate into renal epithelial cells [18, 19]. Recent studies have shown that MSC-derived microvesicles or exosomes mediate the protective effect of MSCs in AKI by promoting the repair of TECs [16, 20].

Exosomes (30–100 nm) are microvesicles (MVs) originated from the multivesicular bodies (MVBs) and can be isolated from diverse body fluids and multiple cell culture supernatants [21-23]. The contents of exosomes are of a complex nature, including various types of proteins, RNAs, enzymes and lipids, which act as messengers during cell-to-cell communication [23, 24]. It has been reported that exosomes derived from MSCs mediate the regenerative process of TECs by transducing RNAs and micro-RNAs into TECs and endothelial cells in AKI models [25-27]. However, almost all these studies focused solely on AKI, we know very little about the potential role of MSCs and exosomes in the prevention of progression from AKI to CKD and the subsequent renal fibrosis.

Sox9 is a transcription factor that belongs to the Sex-determining region Y box family and plays a crucial role in the development of multiple tissues and organs including kidney [28-31]. It was confirmed that combined mutants of Sox8 and Sox9 in mice led to severe renal dysplasia [32]. Recent translating ribosome affinity purification (TRAP) studies showed that Sox9 expression upregulated significantly within the injured tubular epithelium in early phase of I/R injury, and this activation persisted on day 28 in spite of the recovery of renal function [32, 33]. Further immunofluorescence research demonstrated that about 40% of Sox9-positive cells were proliferating and expanding following renal injury, suggesting that Sox9 might facilitate the intrinsic repair process of injured renal TECs [32]. However, these previous research did not concern the association of Sox9 with MSC treatment in AKI-CKD transition, both of which could promote the repair of renal tubules. Thus, we supposed that hAD-MSC treatment can activate tubular Sox9, thereby mitigating AKI and subsequent chronic kidney fibrosis via tubular dependent exosome shuttling. To verify this hypothesis, we injected hAD-MSCs or exosomes to mice subjected to unilateral I/R and detected the levels of Sox9. Drugs were injected to mice to inhibit the release of exosomes from hAD-MSCs or inhibit the expression of Sox9 in TECs.

## RESULTS

### The hAD-MSCs attenuated murine AKI

**Figure 1:**
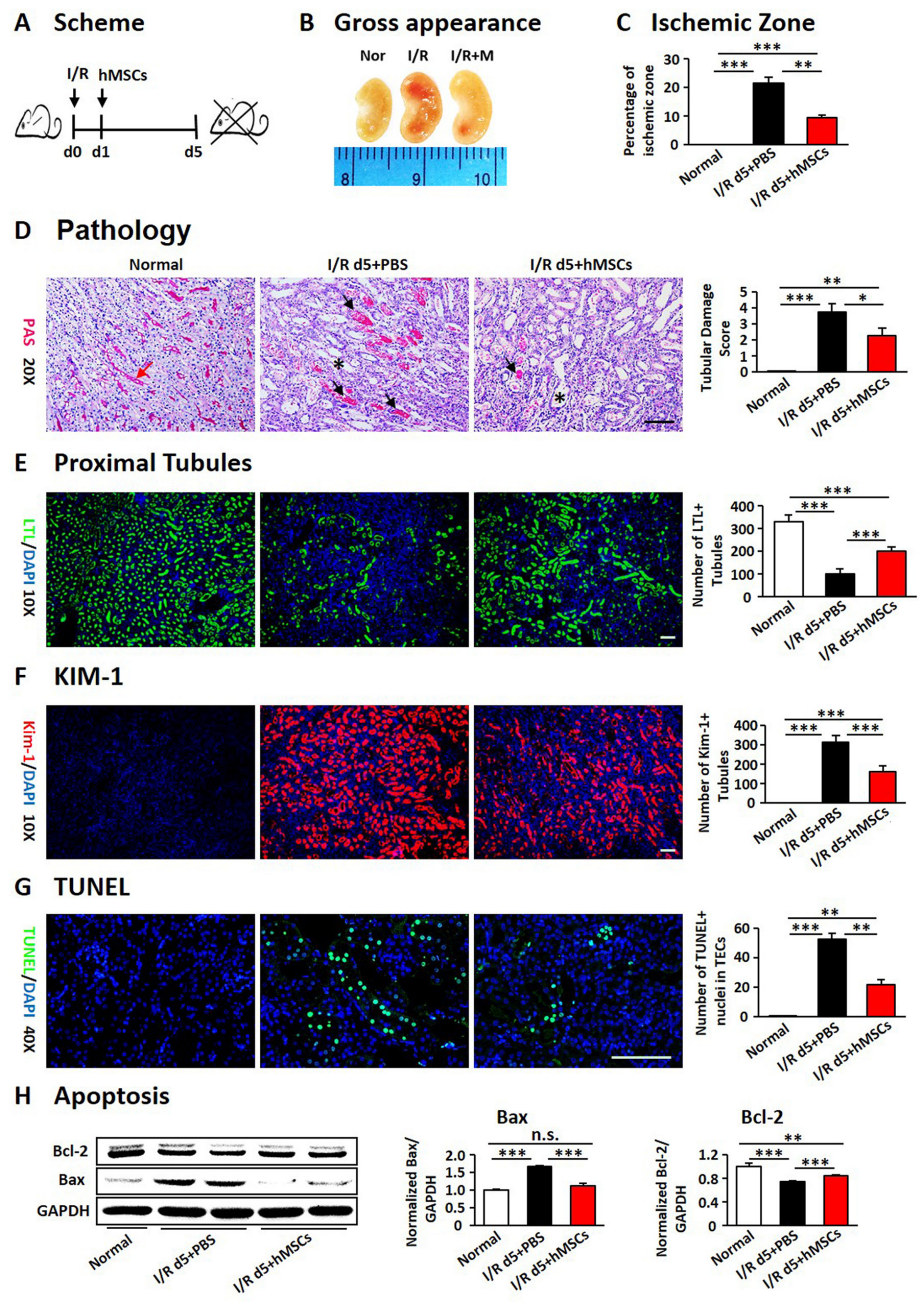
hAD-MSCs alleviated murine AKI in I/R **(A)** Scheme of the experimental plan. **(B)** Kidney gross appearance from mice in each group. **(C)** Analysis of ischemic zone. **(D)** Periodic Acid-Schiff staining and the analysis of renal damage score. Red arrow: proximal tubular brush border; Asterisk: expanded tubules; Black arrow: casts. **(E)** LTL (green) identified proximal tubules. **(F)** KIM-1 (red) represented injured tubules. Original magnification×100. TUNEL positive nuclei in TECs by immunofluorescence **(G)**. Original magnification×400. Expression of Bax and Bcl-2 by Western Blotting **(H)**. Scale=100 μm. N=5/group. Values were means ±SEM. *P < 0.05, **P < 0.01, ***P<0.001.

In this study, hAD-MSCs from patients underwent liposuction surgery were isolated and were cultured in plastic flasks (Supplementary Figure 1A). The cells were positive for mesenchymal markers of CD105, CD44 and CD90, and were negative for CD34 (a mark for hematopoietic cells) or CD45 and CD11b (markers for myeloid cells) (Supplementary Figure 1B). The hAD-MSCs were transferred to mice through tail vein on day 1 after I/R injury (Figure 1A). An obvious ischemic zone was seen at the junction of cortex and medulla in injured kidneys when sacrificed on day 5, which was significantly reduced after hAD-MSC treatment (Figure 1B and 1C). I/R induced tubular epithelial cell edema, necrosis, tubular dilation, casts formation and brush border loss, which were significantly alleviated with hAD-MSC treatment (Figure 1D). To fully compare the loss of tubules, we used lotus tetragonolobus lectin (LTL) to detect proximal tubules. As shown in Figure 1E, the LTL-positive tubules were restored compared to the injured kidney after hAD-MSC treatment. The kidney injury molecule 1 (Kim-1) was less expressed in hAD-MSC group compared with non-treated I/R mice (Figure 1F). We also observed that TUNEL-positive TECs were reduced after hAD-MSC treatment, accompanied by the increasing expression of Bcl-2 and the decreasing expression of BAX (Figure 1G and 1H). We did not observe any difference between the cell injection group (without I/R) and the control group (data not shown). We also examined renal function and found no significant change between I/R and hAD-MSC treated group (Supplementary Figure 2A). However, hAD-MSC therapy did improve renal function in folic acid nephropathy (Supplementary Figure 2B). These data suggestedthat hAD-MSC treatment alleviated murine AKI.

### The hAD-MSCs promoted regeneration of TECs

**Figure 2:**
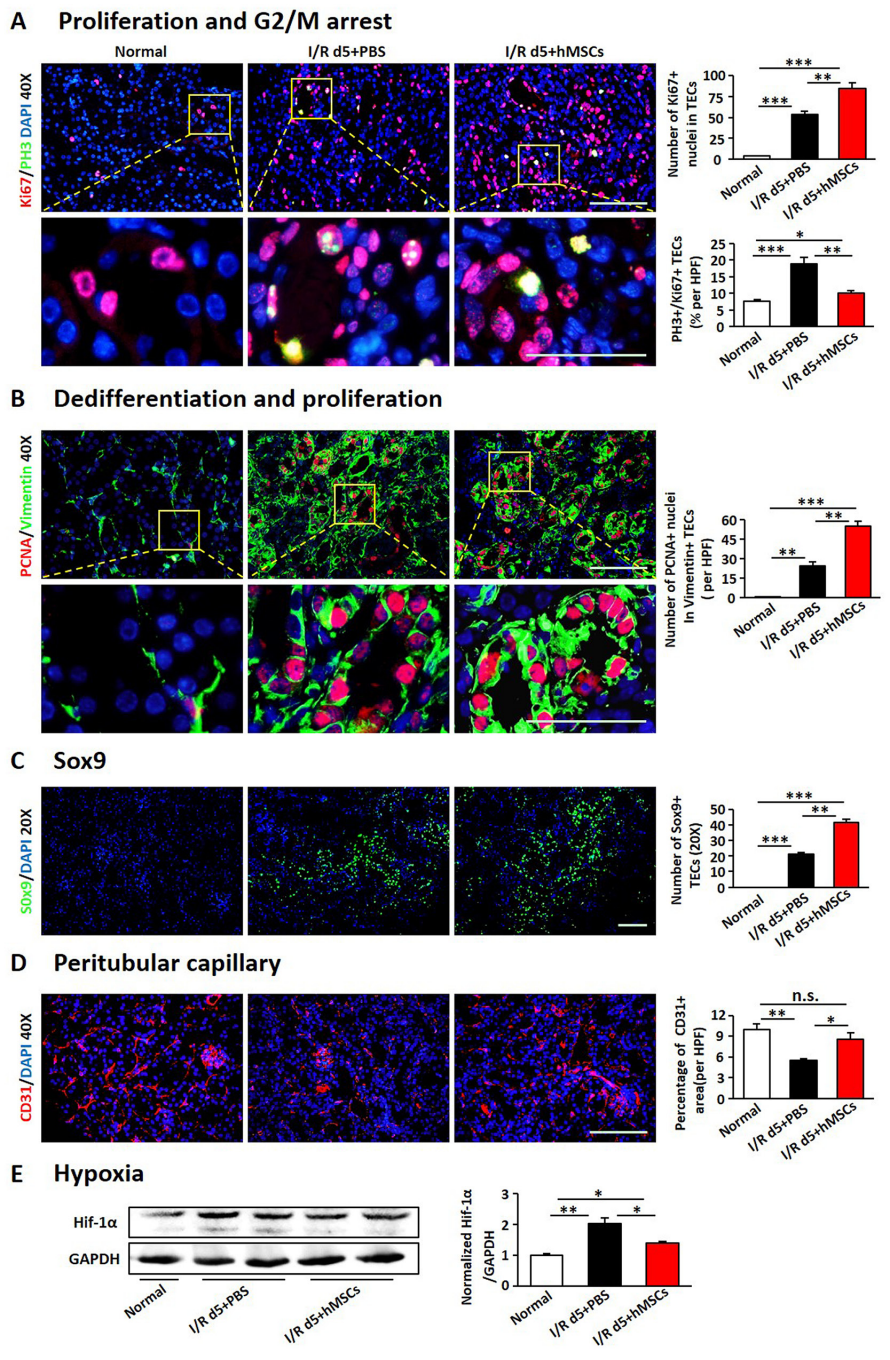
hAD-MSCs promoted regeneration of TECs **(A)** Co-localization of Ki67 (red) and PH3 (green) after I/R. **(B)** Co-localization of PCNA (red) and Vimentin (green) after I/R. Original magnification×400. **(C)** Sox9 expression (green) by immunofluorescence. Original magnification×200. **(D)** Peritubular capillary density assessed by CD31 immunofluorescence. Original magnification×400. **(E)** Hypoxia was assessed by immunoblotting against Hif-1α. TECs: tubular epithelial cells. Scale=100 μm. N=5/group. Values were means ±SEM. *P < 0.05, **P < 0.01, ***P<0.001.

Previous studies have shown that AKI accompanished with regeneration and repair of renal TECs [19, 34]. We found I/R injury induced a slight increase in Ki67-positive nuclei in TECs, which significantly increased in hAD-MSC treated group (Figure 2A), suggesting that hAD-MSC treatment promoted proliferation of TECs. However, proliferation doesn’t mean survived and regeneration of TECs. Effective proliferation in TECs is characterized as with the capacity to dedifferentiate and proliferate, called “self-duplication model” [35]. To make it clear, we performed immunofluorescence double labeling with antibodies against PCNA and vimentin, whose positive in TECs means dedifferentiation [36]. As shown in Figure 2B, hAD-MSC treatment increased the proportion of PCNA and vimentin double positive cells than non-treated I/R group, suggesting that hAD-MSC promoted the regeneration of injured TECs.

As described previously that cell cycle arrest in TECs mediates kidney fibrosis after AKI [37, 38], we next performed immunofluorescence double labeling with antibodies against Ki67 and PH3 to assess the level of cell cycle arrest in TECs. It showed that more than 15 percentage of TECs was double stained with Ki67 and PH3, indicating these cells were arrested in G2/M on day 5 after I/R injury, which was reduced after hAD-MSC treatment (Figure 2A), suggesting that TECs mediated fibrosis was decreased after hAD-MSC treatment compared to non-treated I/R group.

Previous research showed that Sox9 was activated within TECs and might facilitate the repair of injured kidneys [32, 39]. We then detected the level of Sox9 in TECs. As shown in Figure 2C, Sox9-positive cells were almost invisible in control kidneys and AKI triggered a slight increase of tubular Sox9, however, hAD-MSC treatment significantly increased the expression of tubular Sox9, suggested that hAD-MSC treatment activated tubular Sox9.

**Figure 3:**
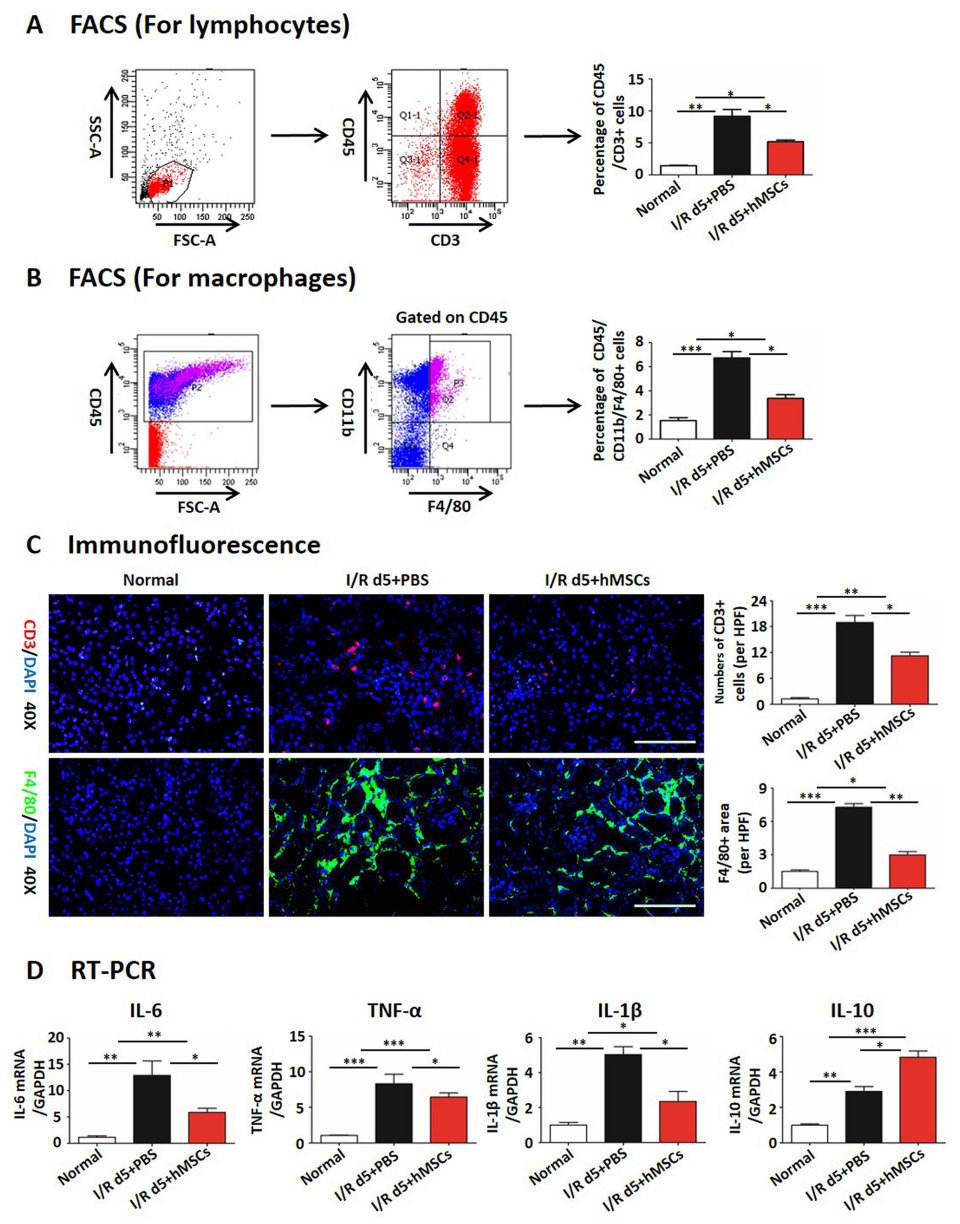
hAD-MSCs reduced infiltration of inflammation Flow cytometry for lymphocytes **(A)** of CD45 and CD3 double positive cells, for macrophages **(B)** of CD45, CD11b and F4/80 triple positive cells. **(C)** Immunofluorescence staining for lymphocytes (CD3, red) and macrophages (F4/80, green). Original magnification×400. Scale=100 μm. **(D)** Secretion of inflammatory cytokines of IL-6, IL-1β, IL-10 and TNF-α in left kidneys by RT-PCR. N=5/group. Values were means ±SEM. *P < 0.05, **P < 0.01, ***P<0.001.

Inflammatory infiltration and peritubular rarefaction are important promoters for the progression of renal injury to CKD [40-42]. We next investigated the effects of hAD-MSC therapy on inflammatory infiltration and peritubular capillary rarefaction. As shown in Figure 2D, I/R injury significantly reduced the percentage of CD31 positive peritubular capillaries, while the percentage was increased after hAD-MSC therapy. The expression of HIF-1α was also reduced in hAD-MSC treated group (Figure 2E), suggesting that hAD-MSC treatment improved kidney hypoxia. hAD-MSC therapy significantly reduced the infiltration of lymphocytes (CD45 and CD3 double positive cells, Figure 3A) and macrophages (CD45, CD11b and F4/80 triple positive cells, Figure 3B) in the interstitium. CD3-positive lymphocytes and F4 / 80-positive macrophages were reduced as confirmed by immunofluorescence in Figure 3C. As shown in Figure 3D, cell therapy also reduced the secretion of inflammatory cytokines induced by I/R and increased anti-inflammatary factor such as IL-10. In summary, hAD-MSC treatment promoted tubular repair and regeneration, through activating tubular Sox9, increasing the dedifferentiation and effective proliferation, improved cell cycle arrest and hypoxia, prevented the infiltration of inflammatory cells, which are the hallmarks for the transtion from AKI to CKD [37, 43], suggesting that hAD-MSCs had the potential to delay AKI and subsequent CKD.

### The hAD-MSCs mitigated subsequent renal fibrosis after I/R

**Figure 4:**
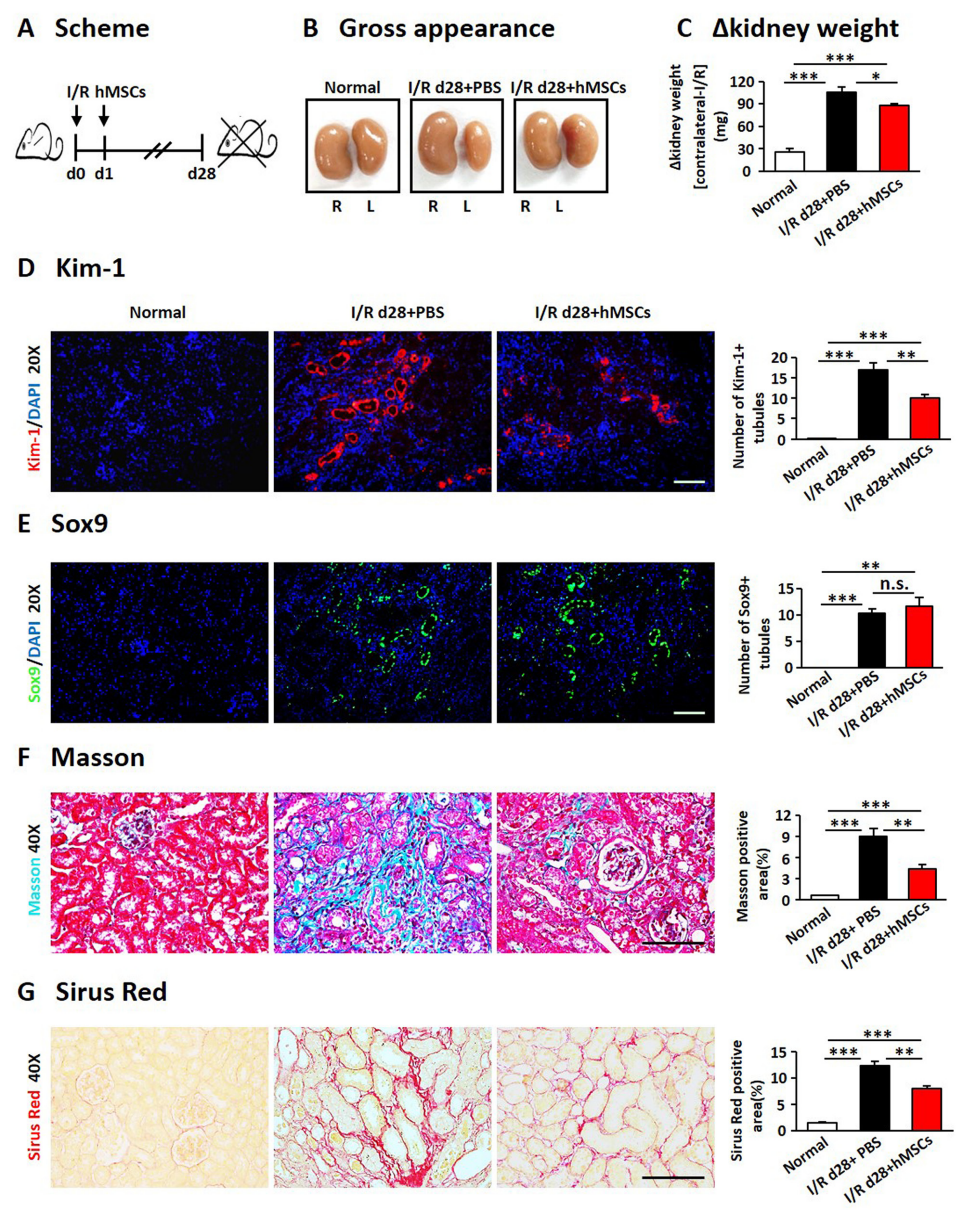
hAD-MSCs mitigated chronic kidney injury in I/R **(A)** Scheme of the experimental plan of chronic renal injury in C57BL/6 mice on day 28. **(B)** Kidney gross appearance from mice in each group. **(C)** Statistical analysis of the change in kidney weights (contralateral minus I/R kidney). KIM-1 (red) **(D)** and Sox9 (green) **(E)** expression immunofluorescence. Original magnification×200. Collagen deposition using Masson **(F)** and Sirus Red **(G)** staining. Original magnification×400. Scale=100 μm. N=5/group. Values were means ±SEM. *P < 0.05, **P < 0.01, ***P<0.001.

**Figure 5:**
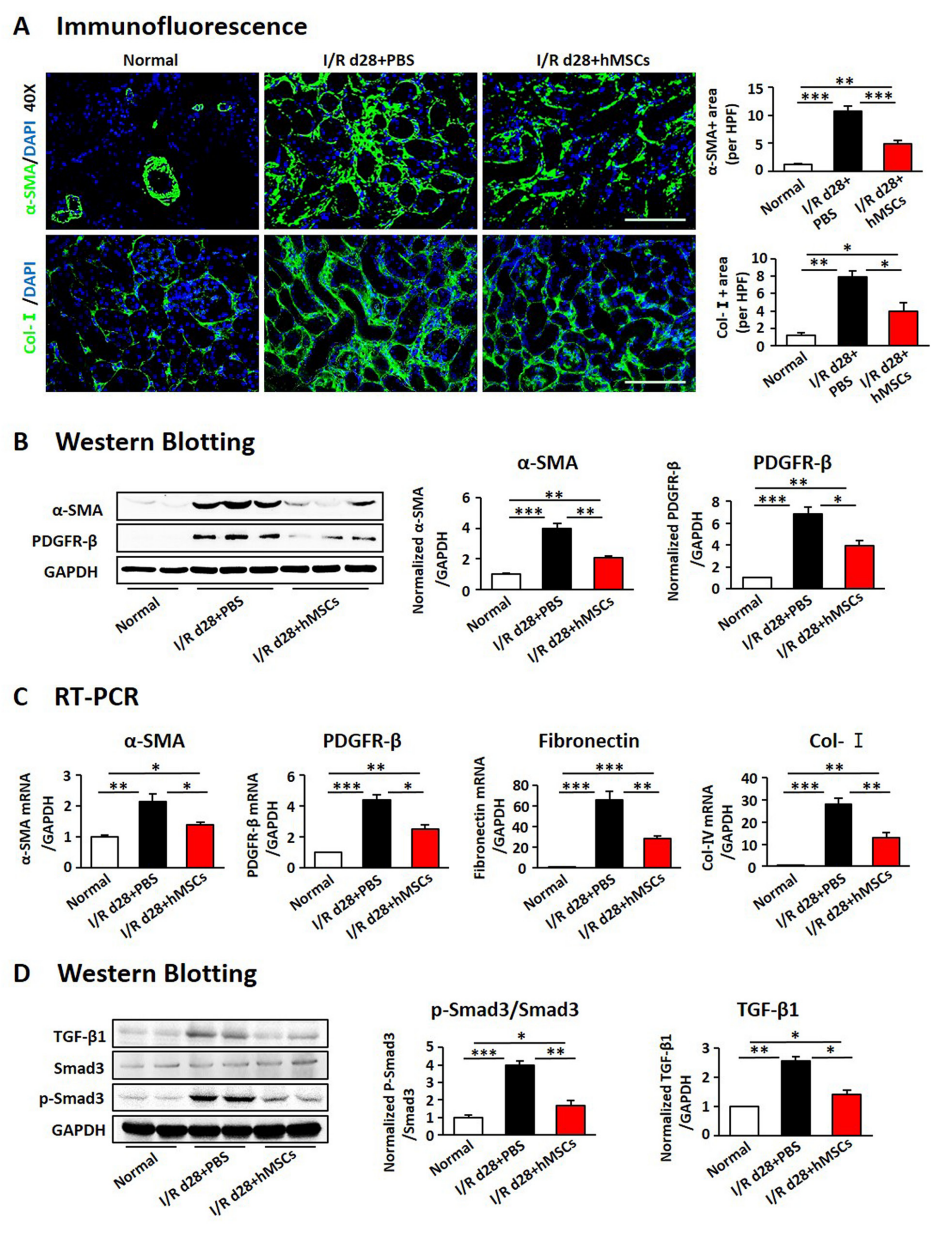
hAD-MSCs attentuated kidney interstitial fibrosis **(A)** α-SMA (green, upper) and Collagen-I (green, lower) expression by immunofluorescence. Original magnification×400. Expression of α-SMA and PDGFR-β using immunoblotting **(B)** and RT-PCR **(C)**. **(D)** Expression of TGF-b1 and Smad3 by Western Blotting. Scale=100 μm. N=5/group. Values were means ±SEM. *P < 0.05, **P < 0.01, ***P<0.001.

We next examined the effects of hAD-MSCs on I/R induced renal fibrosis (Figure 4A). Kidney weight and size were more dramatically decreased in non-treated I/R group compared to hAD-MSC treatment group on day 28 after I/R (Figure 4B and 4C). Kim-1 was also reduced in hAD-MSC treatment group (Figure 4D). As shown in Figure 4E, Sox9 was still activated in TECs on day 28 and there were no significant differences between I/R and hAD-MSC treatment kidneys. Masson (Figure 4F) and Sirius Red (Figure 4G) staining showed the collagen deposition was reduced in renal interstitium after hAD-MSC treatment compared to the control group. As the activation and proliferation of myofibroblasts is a key step in the development of renal fibrosis [11, 12, 44], we then investigated the expression of proteins and genes associated with myofibroblasts. As shown in Figure 5A, the expression of specific fibrosis markers α-SMA and Col-I was significantly reduced after hAD-MSC treatment compared to non-treated I/R kidneys. This was also confirmed by Western Blot (Figure 5B) and RT-PCR (Figure 5C). Previous reports suggest that TGF-β1/Smad2/3 is one of the most classical signaling pathways involved in the activation of myofibroblasts in organ fibrosis [45]. Thus, we examined the effects of hAD-MSCs on this pathway. As shown in Figure 5D, we found that hAD-MSC treatment significantly reduced the expression of TGF-b1 in left kidneys and decreased the phosphorylation level of Smad3 compared to non-treated I/R kidneys. Thus, it indicated that hAD-MSC therapy delayed the subsequent renal interstitial fibrosis through inhibiting the activation of TGF-β1/Smad 3 signaling pathway.

### The hAD-MSCs protected injured kidneys through exosome shuttling

**Figure 6:**
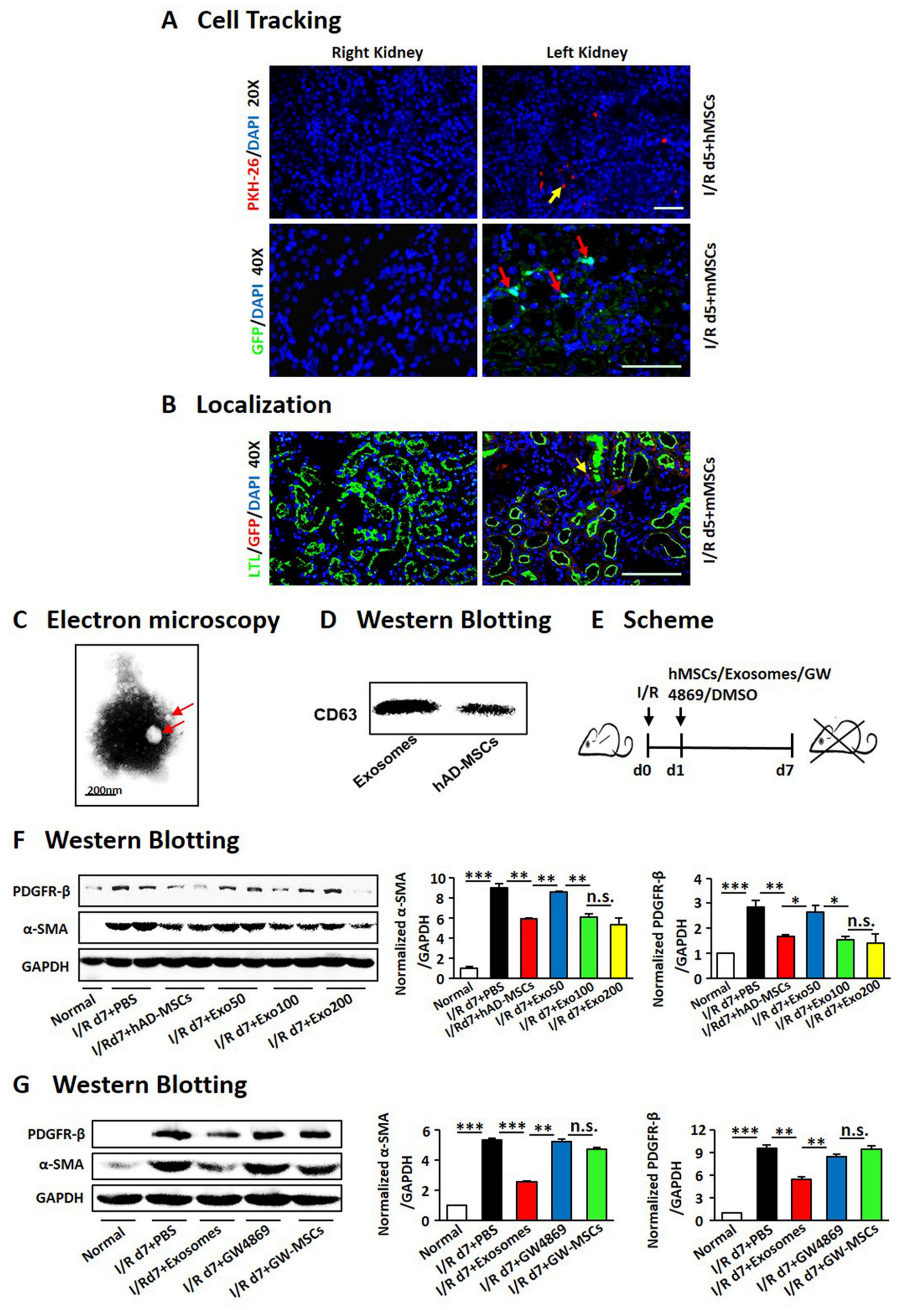
hAD-MSCs protected injured kidneys through exosome shuttling Cell tracking by PKH-26 (red) staining and GFP (green) stainging **(A)**. Original magnification×200 (upper) and ×400 (down). **(B)** Co-staining for LTL (green) and GFP (red) after I/R. Original magnification×400. Characters of exosomes by Electron microscopy **(C)** and Western Blotting **(D)**. **(E)** Scheme of the experimental plan. Concentration gradients after hAD-MSC and exosome treatment **(F)**. Expression of α-SMA and PDGFR-β using immunoblotting **(G)** after exosome and GW4869 treatment. GW4869: a drug inhibiting the release of exosomes. Scale=100 μm. N=5/group. Values were means ±SEM. *P < 0.05, **P < 0.01, ***P<0.001.

As fate tracing studies showed the engraftment of MSCs without a direct differentiation into mature TECs [46, 47], we tried to confirm it by using several methods to trace the injectedhAD-MSCs. Firstly, hAD-MSCs were marked with PKH-26 (Figure 6A), and we observed only a few labeled cells in kidneys. We then extracted AD-MSCs from GFP mice and injected them into I/R mice (Figure 6A). It also showed very little cells in the interstitium of injured kidneys. As shown in Figure 6B, we didn’t find out any GFP-positive cells in LTL-positive tubules, confirming that the injected AD-MSCs did not directly differentiate into renal tubular cells.

Hence we further explored whether exosomes derived from hAD-MSCs benefit kidneys. Exosomes from hAD-MSCs were purified and identified by transmission electron microscopy (Figure 6C), under which a homogenous pattern of spheroid particles with membrane structure was identified. By Western Blot, exosomes derived from hAD-MSCs were positive for CD63 (Figure 6D), a marker for membranes of intracellular vesicles. Those exosomes were injected into mice suffering from I/R through tail vein at different concentrations 24 hours after reperfusion (Figure 6E). Kidneys were obtained on day 7. It showed that exosomes derived from hAD-MSCs attenuated kidney injury at the dose of 100 ug (Figure 6F). In Figure 6G, exosome treatment reduced expression of myofibroblast markers, α-SMA and PDGFR-β. At the same time, the hAD-MSC-induced attenuation of renal fibrosis was reversed after GW4869 treatment (Figure 6G). Thus, hAD-MSCs ameliorated AKI and subsequent renal fibrosis through paracrine of exosomes.

### Sox9 was activated in TECs after hAD-MSC and exosome treatment

**Figure 7:**
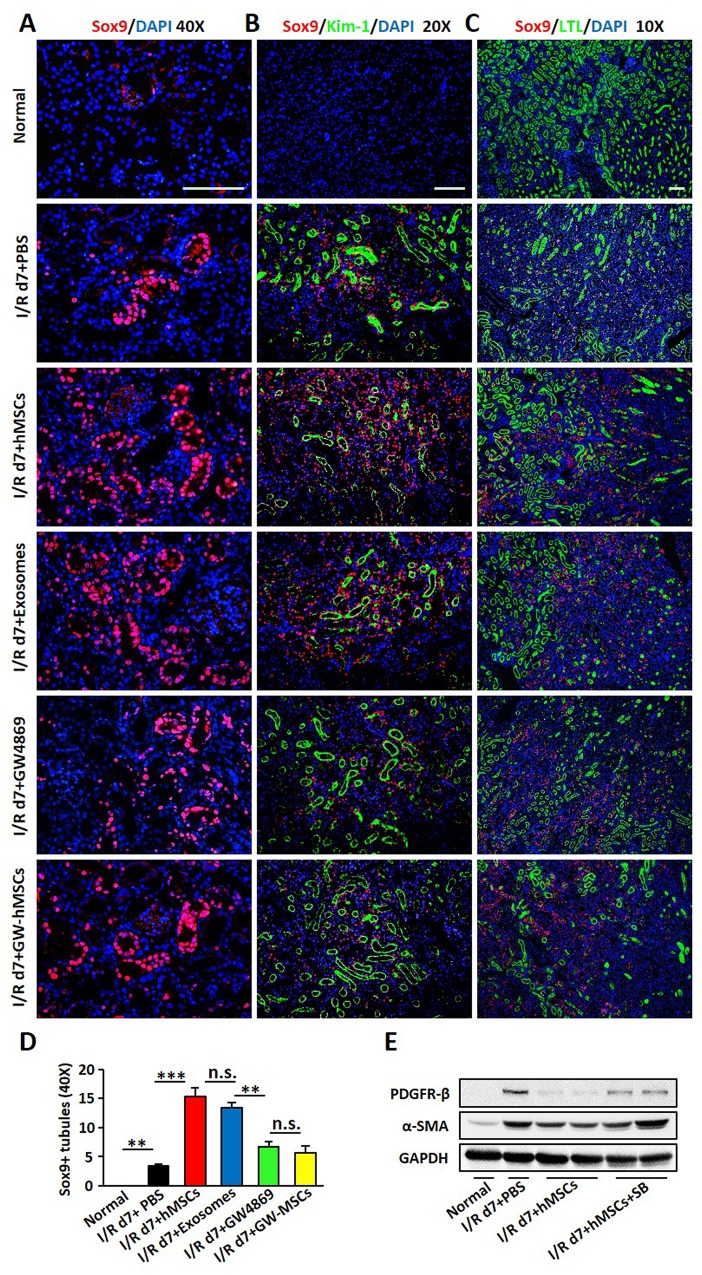
Sox9 was activated in TECs after hAD-MSC and exosome treatment **(A)** Sox9 expression in TECs by immunofluorescence 7 days after I/R. Original magnification×400. **(B)** Co-staining of Sox9 (red) and Kim-1 (green) expression. Original magnification×200. **(C)** Co-localization of Sox9 (red) and LTL (green) expression. Original magnification×100. Scale=100 μm. **(D)** Quantitative analysis of Sox9 in (A). **(E)** Expression of α-SMA and PDGFR-β after SB203580 treatment. SB: SB203580, an inhibitor for Sox9. N=5/group. Values were means ±SEM. *P < 0.05, **P < 0.01, ***P<0.001.

Sox9 has been reported to be the most highly upregulated gene in TECs upon AKI [32, 33], we next explored the profile of Sox9 after hAD-MSC treatment. We found that Sox9 was activated slightly in I/R group, but was dramatically activated in tubular epithelium after hAD-MSC or exosome treatment on day 7 of I/R injury, which was reversed by GW4869 inhibiting the release of exosomes (Figure 7A). To examine Sox9 response in detail, we then conducted immunofluorescence double labeling with antibodies against Sox9 and LTL or Kim-1. As shown in Figure 7B and 7C, none Sox9 or Kim-1-positive tubules were found in normal kidneys, in which numerous LTL-positive tubules were seen. We observed more LTL and Sox9-positive tubules after hAD-MSC and exosome treatment than I/R and GW4869 treated mice (Figure 7D), suggesting that Sox9 activation may facilitate kidney regeneration. We also found more Kim-1-positive tubules in I/R and GW4869 treated mice in Figure 7B, only some of these tubules were Sox9-positive. Less Kim-1-positive tubules were observed in hAD-MSC and exosome treatment group, however, almost all these tubules were Sox9-positive, suggesting that Sox9 activation mitigated kidney injury. We next used an inhibitor of Sox9, SB203580. The expression of Sox9 was reduced after drug treatment (data now shown), however, the expression of α-SMA and PDGFR-β were increased in I/R group compared to hAD-MSC therapy group (Figure 7E).

### The hAD-MSCs employed exosomes to prevent the transformation of TECs, not fibroblasts, into pro-fibrotic phenotype via activating tubular Sox9

**Figure 8:**
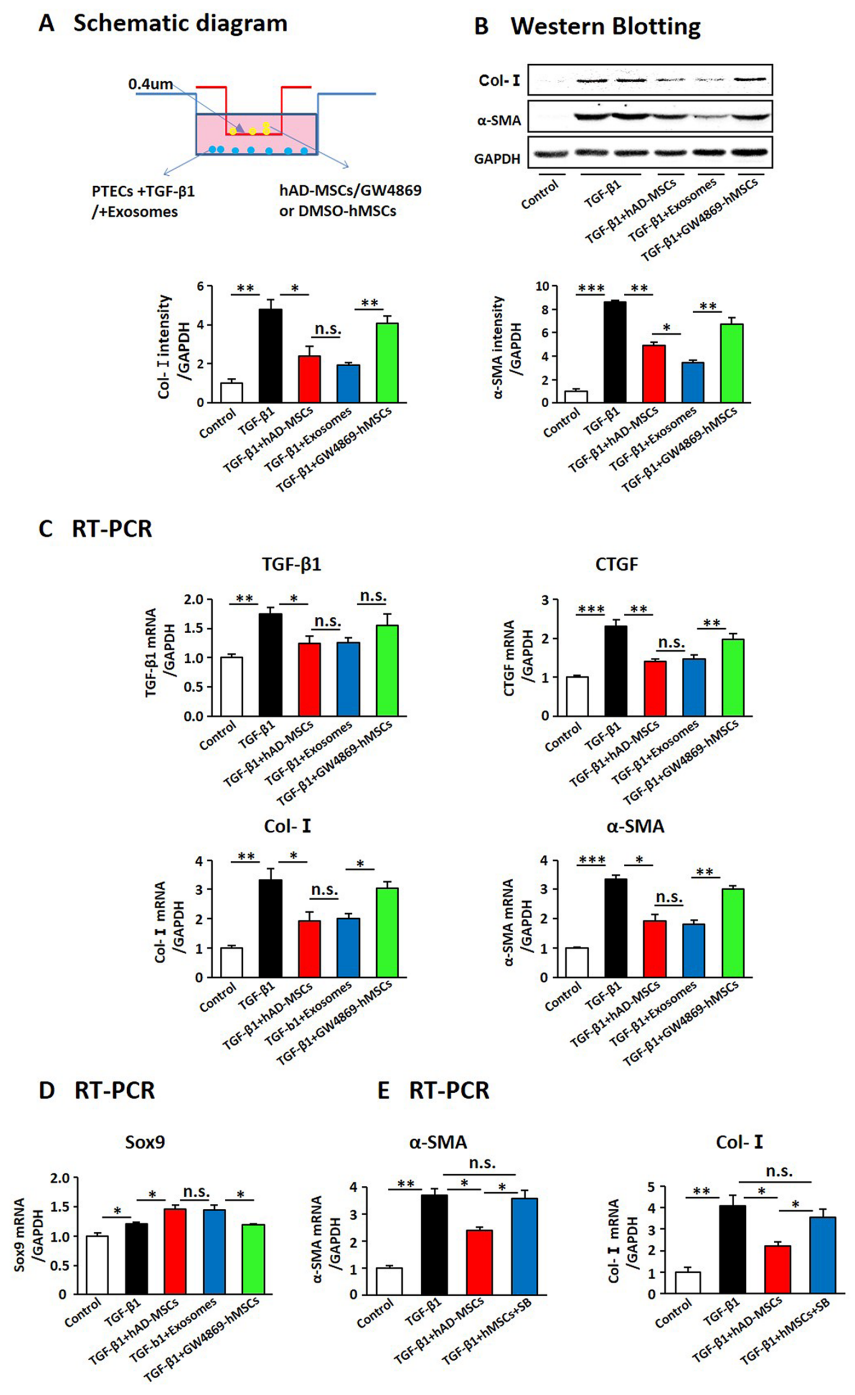
hAD-MSCs prevented the transformation from TECs to pro-fibrotic phenotype induced by TGF-β1 through exosome shuttling **(A)** Scheme of the *in vitro* experimental plan. Expression of fibrosis associated factors by West Blotting **(B)** and RT-PCR **(C)**. Expression of Sox9 in TECs using RT-PCR **(D)**. **(E)** Expression of α-SMA and Col-I by RT-PCR after inhibiting Sox9 in TECs. SB: SB203580, an inhibitor for Sox9. N=5/group. Values were means ±SEM. *P < 0.05, **P < 0.01, ***P<0.001.

Previous studies demonstrated that kidney injury triggers a senescence-associated profibrotic secretory phenotype of TECs and subsequent renal fibrosis [38, 48]. Thus we investigated the effect of hAD-MSCs on TGF-β1 induced transformation of TECs into a pro-fibrotic phenotype. After 24 hours of TGF-β1 exposure, the primary TECs were co-cultured with hAD-MSCs or exosomes in transwell chambers with or without GW4869 for another 24 hours (Figure 8A). As shown in Figure 8B and 8C, the expression levels of α-SMA, Col-I, TGF-β1 and CTGF decreased after hAD-MSC and exosome treatment, but were reversed by inhibiting the release of exosomes from hAD-MSCs. Sox9 expression in TECs was increased upon hAD-MSC treatment but decreased after inhibiting the release of exosomes from hAD-MSCs in Figure 8D. As shown in Figure 8E, the expression levels of α-SMA and Col-I were increased after inhibiting Sox9 in TECs. It suggested that hAD-MSCs prevented TGF-β1 induced transformation of TECs into pro-fibrotic phenotype via exosome shuttling between hAD-MSCs and TECs, accompanied by tubular Sox9 activation.

**Figure 9:**
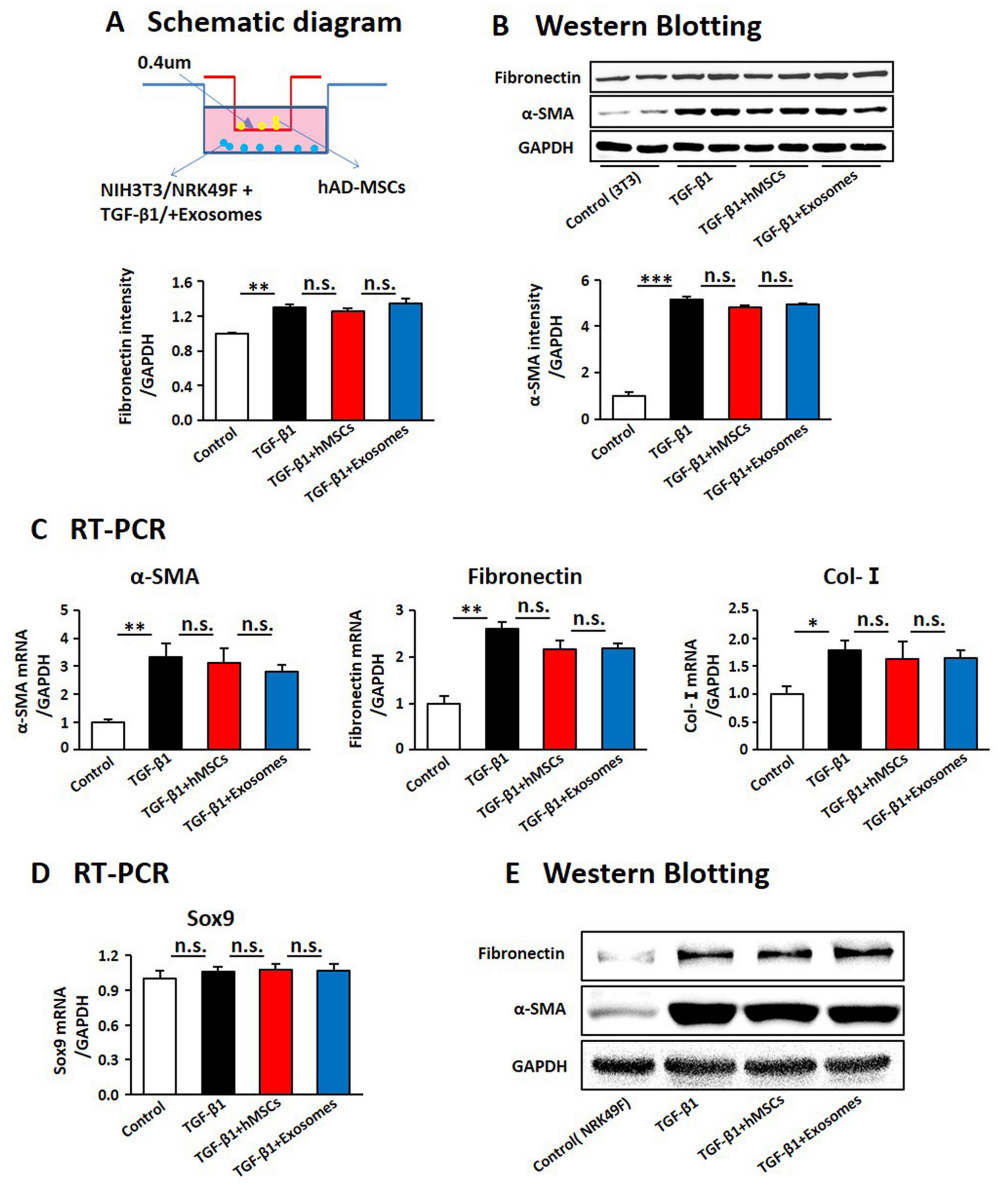
hAD-MSCs and exosomes had no significant effect on the activation of embryonic fibroblasts induced by TGF-β1 **(A)** Scheme of the *in vitro* experimental plan. Expression of factors associated with the activation of NIH3T3 by West Blotting **(B)** and RT-PCR **(C)**. Expression of Sox9 in NIH3T3 by RT-PCR **(D)**. **(E)** Expression of α-SMA and fibronectin by Western Blotting in NRK49F. NIH 3T3: a cell line of mouse embryonic fibroblast. NRK49F: a cell line of rat kidney fibroblast. N=5/group. Values were means ±SEM. *P < 0.05, **P < 0.01, ***P<0.001.

As activation of myofibroblasts is the key point in the progression of renal fibrosis [11, 12], we evaluated whether hAD-MSCs directly inhibit TGF-β1 induced activation of fibroblasts into myofibroblasts (Figure 9A). It showed that the expression levels of α-SMA and fibronectin were increased after TGF-β1 treatment, suggesting these cells were activated into myofibroblasts. However, it did not show a decrease of α-SMA and fibronectin after co-cultured with hAD-MSCs and exosomes (Figure 9B and 9C), which suggested that hAD-MSCs and exosomes did not directly inhibit the transformation of fibroblasts into myofibroblasts. We didn’t observe changes of Sox9 expression among each group in Figure 9D. We detected the same phenomenon in NRK49F, a cell line of rat kidney fibroblast (Figure 9E). Thus, hAD-MSCs prevented TGF-β1 induced transformation of renal TECs, but not fibroblasts, into pro-fibrotic phenotype via exosome shuttling accompanied by Sox9 activation, which in turn delayed the progression of AKI into CKD and kidney fibrosis.

## DISCUSSION

We report here the finding that hAD-MSCs employed exosomes to attenuate murine AKI to CKD progression through tubular epithelial cell dependent Sox9 activation in model of unilateral renal I/R injury. The principle findings were that hAD-MSC treatment reduced renal pathological damage, promoted effective proliferation of TECs, improved cell cycle arrest of TECs, reduced renal ischemia and hypoxia and decreased the infiltration of inflammatory cells. The hAD-MSC treatment also alleviated subsequent renal fibrosis and inhibited transformation of TECs, but not fibroblasts into a pro-fibrotic phenotype induced by TGF-β1. The mechanism of hAD-MSCs mitigating AKI to CKD progression lied in the exosome shuttling into TECs, thereby activating tubular Sox9, which was reversed by a drug blocking the release of exosomes from hAD-MSCs or a drug inhibiting the expression of Sox9 in TECs.

Exogenous stem cell therapy has been widely used to treat ischemic diseases as an adjunctive strategy in the past few decades [49], however its mechanism is still unclear. Here, we have demonstrated that infusion of human AD-MSCs into I/R mice conferred profound renalprotection to the injured kidneys not only in acute phase also in subsequent chronic phase. Sox9 has been reported to be an early injury response signature within the kidney epithelium and take part in the intrinsic mechanisms regulating kidney development and repair [31, 32, 39, 50], however previous research did not associate hAD-MSCs with Sox9 activation, both of which had been reported to facilitate regeneration of injured kidneys. In this study, we have proved that hAD-MSCs employed exosomes to attenuate murine AKI to CKD progression through tubular epithelial cell dependent Sox9 activation.

In contrast to previous reports, we have demonstrated that hAD-MSC therapy promoted efficient proliferation of TECs, which suggested that the survival cells from injury performed dedifferentiation and proliferation, and then redifferentiated into proximal tubular epithelium. We further reported that hAD-MSCs treatment changed hallmarks of AKI-CKD transition, manifestied by improved tubular cell cycle arrest in G2/M phase, reduced renal hypoxia and decreased the infiltration of inflammatory cells into kidney interstitium. Li Yang and colleagues [38] verified that TECs arrested in G2/M phase could not perform normal mitosis, seriously preventing the repair and regeneration of TECs, which promotes the progression of AKI. Sox9 activation has been proved to highlight an intrinsic cellular pathway of tubular regeneration in AKI [32, 50]. hAD-MSC treatment further promoted the activation of tubular Sox9, which synthetically facilitated the repair and regeneration of TECs through advancing the time point of proliferation and repair, and reducing the proportion of cells in aberrant cell cycle phase and improving renal hypoxia. Eirin A and colleagues used a porcine model of metabolic syndrome and renal artery stenosis and confirmed that extracellular vesicles derived from mesenchymal stem cells mitigated renal inflammation, which was mediated by IL-10 [51]. In our study, we also demonstated that hAD-MSC therapy attenuated the infiltration of inflammatory cells and increased the anti-inflammatory factor--IL-10, suggested that hAD-MSC therapy modified the inflammatory milieu of kidney. The ischemic, hypoxic and inflammatory microenvironment have been effectively improved, which not only reduced acute kidney injury, also attenuated chronic fibrosis, thereby improving renal prognosis.

Our studies confirmed that hAD-MSC treatment alleviated chronic renal injury and fibrosis, as had been proved already in some previous research in models of unilateral ureteral obstruction [52] and folic acid [17] induced kidney fibrosis. However, unlike previous studies, we used the model of unilateral renal ischemia-reperfusion without contralateral nephrectomy, which had been confirmed as a robust model for the study of AKI-CKD progression by both histology and gene expression [53]. Furthermore, we verified that co-cultured TECs with hAD-MSCs and exosomes *in vitro* activated Sox9 and reduced TGF-β1 induced expression of proteins and genes associated with fibrosis. Sox9 activation in renal TECs has been proved to reduce fibrosis and genetic knockout of tubular Sox9 aggravated kindey injury and subsequent fibrosis [50]. In our study, inhibiting the expression of Sox9 reversed the beneficial effects of hAD-MSCs, which was consistent with previous research.

TECs have been identified to uptake exosomes and subsequently change the phenotype of epithelial cells by reducing the release of fibrosis-associated factors, while depleting the microRNAs in MSCs and extracellular vesicles obviously inhibited the intrinsic regenerative potential of epithelial cells [16]. Our immunofluorescence results confirmed that Sox9 was expressed only in TECs, suggesting there might exist a specific receptor for Sox9 on TECs, thus, we assumed that hAD-MSCs might employ exosomes to deliver some genetic materials of Sox9 to TECs, or activate the tubular receptors of Sox9. Thus, the transcription and translation of Sox9 were activated in injured TECs and then synthetically promoted regeneration of injured kidneys. However, we found that co-cultured NIH3T3 or NRK49F with hAD-MSCs and exosomes did not reduce TGF-β1 induced activation of those two kinds of cells. We did not observe changes in Sox9 expression in NIH3T3 and NRK49F. The crosstalk between cells is relatively specific. Thus, it is likely to speculate that hAD-MSCs used exosomes to act directly on TECs by activating tubular Sox9, not only transmitting anti-fibrotic factors, also reducing the secretion of pro-fibrotic factors from injured TECs. Its effect on fibroblasts *in vivo* might be indirect, which was accomplished by reducing the secretion of pro-fibrotic factors of TECs, subsequently inhibiting the activation of fibroblasts.

In this study, human AD-MSCs were used to treat murine renal injury aiming to eliminate the possibility of human stem cells differentiating into mouse renal TECs. Combining the finding that few injected cells were remained in the injured kidneys, we speculated that the protective effect of hAD-MSCs was achieved by paracrine action, not depending on differentiating into renal parenchymal cells. This hypothesis was confirmed by the results that the exosomes derived from hAD-MSCs could exert equivalent protective role as cells. This was supported further by our findings that inhibition the release of hAD-MSC-derived exosomes reversed the beneficial effect. These findings were consistent with previous research demonstrating that MSCs reduced AKI through paracrine [20, 54], rather than directly differentiating into renal parenchymal cells. With the development of exosomes in the field of tumors [23], it was hypothesized that stem and progenitor cells can secrete exosomes to exert organ protective effects. This hypothesis was verified in studies showing that exosomes extracted from MSCs had similar protective efficacy, which was inhibited by blocking the producing of exosomes and other microparticles [55]. Our studies showed that exosomes extracted from hAD-MSCs exerted renal protective effects and was reversed by drugs inhibiting the release of exosomes, which was consistent with reports in other organs [55, 56]. Exosomes are membranous structures with small diameters, which allows free shuttle between cells, and carry important genetic materials from the source cells to transfer them to target cells [21]. In our study, exosomes may carry mRNAs, miRNAs or proteins of Sox9 and deliver them to TECs, thus activated the receptors of Sox9 on TECs and promoted regeneration of injured tubules. We will clarify the specific mechanism of the activation of Sox9 in future studies.

In summary, our research indicated that hAD-MSC therapy employed exosomes to reduce AKI and subsequent CKD progression through tubular epithelial cell dependent Sox9 activation. Inhibiting the release of exosomes or the expression of Sox9 abolished the beneficial effects.

## MATERIALS AND METHODS

### Adipose-derived mesenchymal stem cells

hAD-MSCs and AD-MSCs from GFP mice (Jackson Laboratory, Bar Harbor, ME, USA) were extracted from human adipose tissue of patients suffered from liposuction surgery and inguinal adipose tissue, respectively. In general, adipose tissue was isolated and washed in PBS and was homogenized and enzymatic hydrolyzed in 0.1% collagenase-I at 37°C for 2 hours in a 5% CO2 incubator with shaking every 15 minutes. Then the digestion was terminated by 3-5 times the volume of collagenase-I of complete medium. The cell suspension was filtered through a 70um cell strainer. The cells were cultured in DMEM/F12 (Gibco, USA) supplemented with 10% FBS (Gibco, USA) and 1% penicillin and streptomycin. The medium was changed after 48 hours and replaced every three days. The cells were used between the third to sixth passages. Stem cells were identified through differentiation assays and Flow Cytometry as previously described [15]. Cell treatment: the third generation of hAD-MSCs were suspended in 200uL sterile PBS and injected into mice with or without I/R injury through tail vein 24 hours after I/R. Control mice were injected with 200uL sterile PBS. In some experiments, mice were administrated with 2.5ug/g GW4869 (Sigma, USA) or equal volume of DMSO intraperitoneally 24hours after I/R. hAD-MSCs were pretreated with 20uM GW4869 or DMSO for 2 hours *in vitro* and then were injected into I/R mice in 200uL sterile PBS. In some other experiments, mice were treated with 50mg/kg SB203580 (MedChemExpresss, USA) to inhibit Sox9 or equal volume of DMSO intraperitoneally 24hours after I/R. hAD-MSCs were injected through tail wein one hour after SB203580 or DMSO treatment.

### Isolation of hAD-MSC-derived exosomes

Exosomes were obtained from the supernatants of hAD-MSCs through ultracentrifugation according to classical methods reported in the literature [57]. hAD-MSCs were cultured overnight in DMEM/F12 with 10% exosome-depleted FBS (Gibco, USA), and the medium was collected. The supernatants were centrifuged at 2000g for 30minutes and filtered through a cell filter of 0.22um to remove debris and the cell-free medium was then centrifuged at 100000g (Beckman Coulter, USA) for 1hour at 4°C. The supernatants were discarded and the pellets were washed in M199, after which the suspension was centrifuged at 100000g for 1hour at 4°C. hAD-MSC-derived exosomes were characterized by transmission electron microscopy and Western Blot analysis for CD63 (Abcam, UK) expression as previously described [24].

### Cell culture and treatment

Kidneys were removed from normal male C57BL/6 mice under sterile conditions, minced and digested with collagenase-I for 1 hour at 37°C. The digestion was terminated with complete medium and the suspension was filtered through two cell strainers of 40um and 70um. Then the suspension was centrifuged at 1200 rpm for 10 minutes. After cracking of red blood cells and washed twice, mTECs were cultured in flasks in DMEM/F12 supplemented with 10% FBS (Gibco, USA), 1% penicillin and streptomycin and other grow factors. After one week, mTECs were stimulated with recombinant mouse TGF-β1 (10 ng/mL, R&D system, USA) for 24 hours and then co-cultured with hAD-MSCs in transwell chambers for another 24 hours. In some experiments, exosomes were added into mTECs which were stimulated with TGF-β1. Then mTECs were lysed to collect proteins and RNA. In some experiments, primary TECs were treated with SB203580 (MedChemExpresss, USA, 100nM) to inhibit Sox9 before the intervention of TGF-β1 or hAD-MSCs or exosomes. NIH3T3, a cell line of mouse embryonic fibroblast, was bought from Procell (Wuhan, China) and was cultured in DMEM supplemented with 10% FBS (Gibco, USA) and 1% penicillin and streptomycin. Cells were seeded in 6-well plates and starved for 24 hours. Then cells were incubated with recombinant mouse TGF-β1 (10ng/mL, R&D system, USA) for 24 hours without serum. NRK49F, a cell line of rat kidney fibroblast, was presented by Professor Gang Xu in our department. The cells were cultured in DMEM supplemented with 10% FBS (Gibco, USA) and 1% penicillin and streptomycin. Cells were seeded in 6-well plates and starved for 24 hours. Then cells were incubated with recombinant TGF-β1 (10ng/mL, Pepro Tech, USA) for 24 hours without serum. In some experiments, cells were co-cultured with hAD-MSCs or exosomes for another 24 hours after TGF-β1 was washed away. All experiments were repeated for three times.

### Animal model

Male C57BL/6 mice (8-10 weeks old, weighing 22-25g, Hua Fukang, Beijing, China) were anaesthetized with 1% sodium pentobarbital solution (0.009 mL/g, Sigma, USA) by intraperitoneal injection. The left renal pedicle was clamped with an atraumatic vascular clip for 30 minutes (Roboz Surgical Instrument Co, Germany) through a flank incision and the left kidney turned purple subsequent to clamping. Clamps were removed after 30 minutes to start reperfusion and the left kidney reverted to red within approximately 10 seconds. The body temperature was controlled at 36.6°C-37.2 °C by a sensitive rectal probe throughout the procedure (FHC, USA). The muscle layer and skin were closed with 4-0 silk sutures. Sham animal models were subjected to a similar surgical procedure without clamping the left kidney pedicle.

### Histology and immunofluorescence

Kidneys were fixed in 4% paraformaldehyde for 24 hours, embedded in paraffin and sliced into sections (2-3um). PAS staining was used to evaluate kidney pathological injury, and Masson and Sirius Red staining were carried out to estimate the extent of tubular-interstitial fibrosis. The tubular damage score was evaluated based on our previous research [58].

For IF analysis, renal sections were subjected to heat antigen retrieval in a microwave. The nonspecific antigens were blocked with serum for 30 minutes at room temperature. The slides were then incubated with specific primary antibodies against Kim-1 (1:1000, R&D system, USA), LTL (1:50, Vector Laboratories, USA), Ki-67 (1:200, Abcam, UK), PH3 (1:300, Abcam, UK), α-SMA (1:100, Abcam, UK), Collagen-I (1:200, Abcam, UK), Sox9 (1:200, Abcam, UK), CD31 (1:100, BD, USA), vimentin (1:100, Abcam, UK), GFP (1:100, Abcam, UK) and PCNA (1:50, Santa Cruz, USA) at 4°C and further developed with fluorescent labeled secondary antibodies for IF. Nuclei were stained with DAPI. TUNEL was stained using a kit according to the manufacturer’s instructions (Roche, Switzerland). Staining was carefully quantified in each slide by capturing eight randomly chosen fields in a blind manner by two experienced renal pathologists and the data was analyzed by Image Pro Plus software (Media Cybernetics, Rockville, MD, USA).

### Kidney function

Blood was harvested through eyeball removal and centrifuged at 3000 rpm for 10 minutes to obtain mice serum. Serum urea and creatinine levels were investigated using colorimetric assays according to the manufacturer’s instructions (Bioassay System, USA).

### Western blotting

Renal tissues were lysed in RIPA lysis buffer containing protease inhibitor. Equal amounts of proteins (50 μg) were loaded and separated by SDS-PAGE. The gel was transferred onto PVDF membranes (Roche, Switzerland). The membranes were blocked with 5% skimmed milk in TBST for 1 hour at room temperature and were then incubated with appropriate primary antibodies against α-SMA (1:2000, Abcam, UK), Collagen-I (1:1000, Abcam, UK), Hif-1α (1:1000, Abcam, UK), Bcl-2 (1:1000, Abclonal, USA), Bax (1:1000, Abclonal, USA), CD63 (1:1000, Abcam, UK), Fibronectin (1:1000, Abcam, UK), TGF-β1 (1:500, Abcam, UK), Smad3 (1:1000, Abclonal, USA), p-Smas3 (1:1000, CellSignalingTech, USA) and PDGFR-β (1:1000, Abcam, UK) at 4°C overnight. The membranes were incubated with HRP-conjugated secondary antibodies and were visualized by enhanced chemiluminescence (ECL, BioRad, USA). The relative expression levels were normalized to those of GAPDH (1:4000, Abbkine, China). The signal intensity of the targeted band was quantified using Image J (NIH, USA).

### Quantitative real time-PCR

Total RNA was extracted from renal tissues using Trizol reagent according to the manufacturer’s instructions (Invitrogen, USA) and one microgram of RNA was reverse transcribed into first strand cDNA using the GoScript reverse transcription system (Promega, USA). Quantitative PCR was conducted using the SYBR master-mix (Qiagen, Germany) on the Roche light 480II. Relative mRNA expression levels were calculated using the 2^−ΔΔCt^ method and were normalized to the expression levels of GAPDH. The primer sequences for mice were shown in Table 1.

**Table 1:** Primer sequences for RT-PCR

Genes	Primers
Mouse GAPDH	5’-AGGTCGGTGTGAACGGATTTG-3 ’5’-GGGGTCGTTGATGGCAACA-3’
Mouse α-SMA	5’-TCGCTGTCAGGAACCCTGAGACG-3 ’5’-ATCATCACCAGCGAAGCCGGC-3’
Mouse Col-Ι	5’-ATGGATTCCCGTTCGAGTACG-3 ’5’-TCAGCTGGATAGCGACATCG-3’
Mouse PDGFR-β	5’-AGGAGTGATACCAGCTTTAGTCC-3 ’5’-CCGAGCAGGTCAGAACAAAGG-3’
Mouse CTGF	5’-GACCCAACTATGATGCGAGCC-3 ’5’-CCCATCCCACAGGTCTTAGAAC-3’
Mouse Fibronectin	5’-TCCACAGCCATTCCTGCGCC-3 ’5’-GTTCACCCGCACCCGGTAGC-3’
Mouse Sox9	5’-CGGAACAGACTCACATCTCTCC-3 ’5’-GCTTGCACGTCGGTTTTGG-3’
Mouse IL-6	5’-GAGGATACCACTCCCAACAGACC-3 ’5’-AAGTGCATCATCGTTGTTCATACA-3’
Mouse IL-1β	5’-GAAATGCCACCTTTTGACAGTG-3 ’5’-TGGATGCTCTCATCAGGACAG-3’
Mouse TNF-α	5’-CCCTCACACTCAGATCATCTTCT-3 ’5’-GCTACGACGTGGGCTACAG-3’
Mouse IL-10	5’-GCTGGACAACATACTGCTAACC-3 ’5’-ATTTCCGATAAGGCTTGGCAA-3’

### Flow cytometry

Mouse kidney tissue was diced and digested with Collagenase-I at 37°C for 1 hour. The suspension was filtered through a 40 um strainer to remove cell pellets and washed in PBS to generate a single cell suspension after lysis of red blood cells (BD, USA). The cells were washed twice with PBS. The third generation of hAD-MSCs were trypsinized and washed twice with PBS. The cells were then incubated with the following fluorescent antibodies and corresponding isotype controls (Cat.Number: 400605, 400611 and 400507, Biolegend, USA) for 30 minutes shielded from light at room temperature: FITC-conjugated anti-CD45, APC-conjugated anti-CD11b, PE-Cy7-conjugated anti-CD3, PE-conjugated anti-F4/80, APC-conjugated anti-CD34, PE-conjugated anti-CD31, FITC-conjugated anti-CD90, APC-conjugated anti-CD44 and PE-conjugated anti-105 (Biolegend, USA). The positive cells were sorted using a BD FACS and the data were analysed using the FlowJo v7.6.3 software.

### Statistical analysis

All results were presented as means ± SEM from at least three separate experiments. Statistical differences between two groups were analysed using the unpaired *t*-test or Mann-Whitney *U* test by GraphPad Prism 5.0. The statistical significance of differences was set at P < 0.05.

## SUPPLEMENTARY MATERIALS FIGURES


