# Supplementary material for: Adipose-derived mesenchymal stem cells employed exosomes to attenuate AKI-CKD transition through tubular epithelial cell dependent Sox9 activation
Source: Oncotarget. 2017 Aug 7;8(41):70707–26. doi: 10.18632/oncotarget.19979 (PMC5642588; doi:10.18632/oncotarget.19979)
Supplement: Supplementary file 1 [file oncotarget-08-70707-s001.pdf]

Adipose-derived mesenchymal stem cells employed exosomes to attenuate AKI-CKD transition through tubular epithelial cell dependent Sox9 activation

SUPPLEMENTARY MATERIALS

Supplementary materials and methods

Male C57BL/6 mice were intraperitoneally injected with vehicle (150 mM NaHCO3) or folic acid (200 mg/kg, Sigma-Aldrich, USA) after adaptive feeding for 1 week. $3x10^5$ hAD-MSCs were injected through tail wein 6 hours after folic acid injection. Blood samples were obtained 72hours later and centrifuged to get serum. Renal function of creatinine and urea nitrogen were detected according to the manufacturer's instructions.

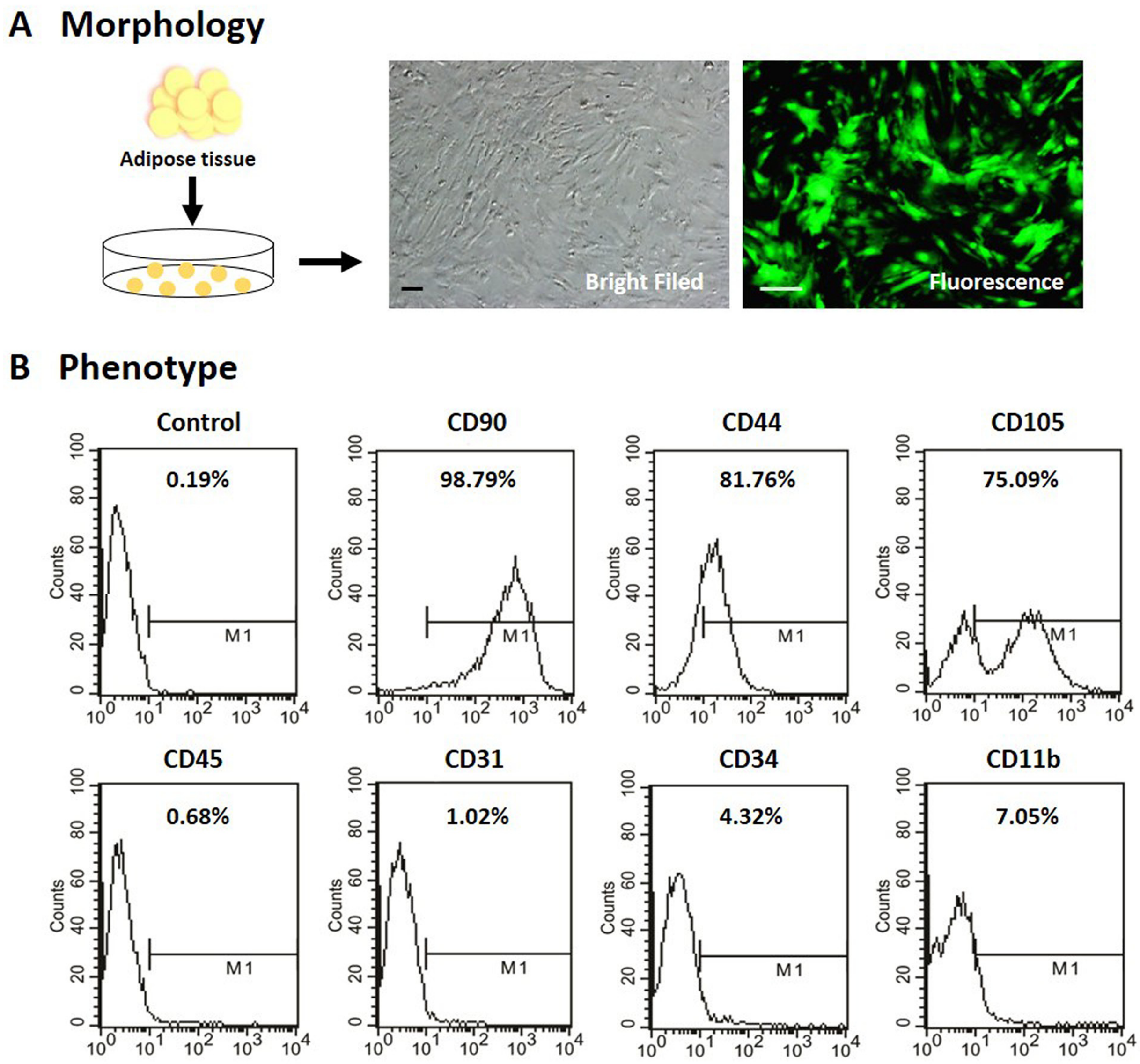

Supplementary Figure 1: Characterization of AD-MSCs. (A) Morphology of the third passage hAD-MSCs under bright field microscopy (left); AD-MSCs from GFP mice under fluorescent microscopy (right). **(B)** Flow cytometry for surface markers of hAD-MSCs. AD-MSCs: adipose-derived mesenchymal stem cells. Scale=100 μm.

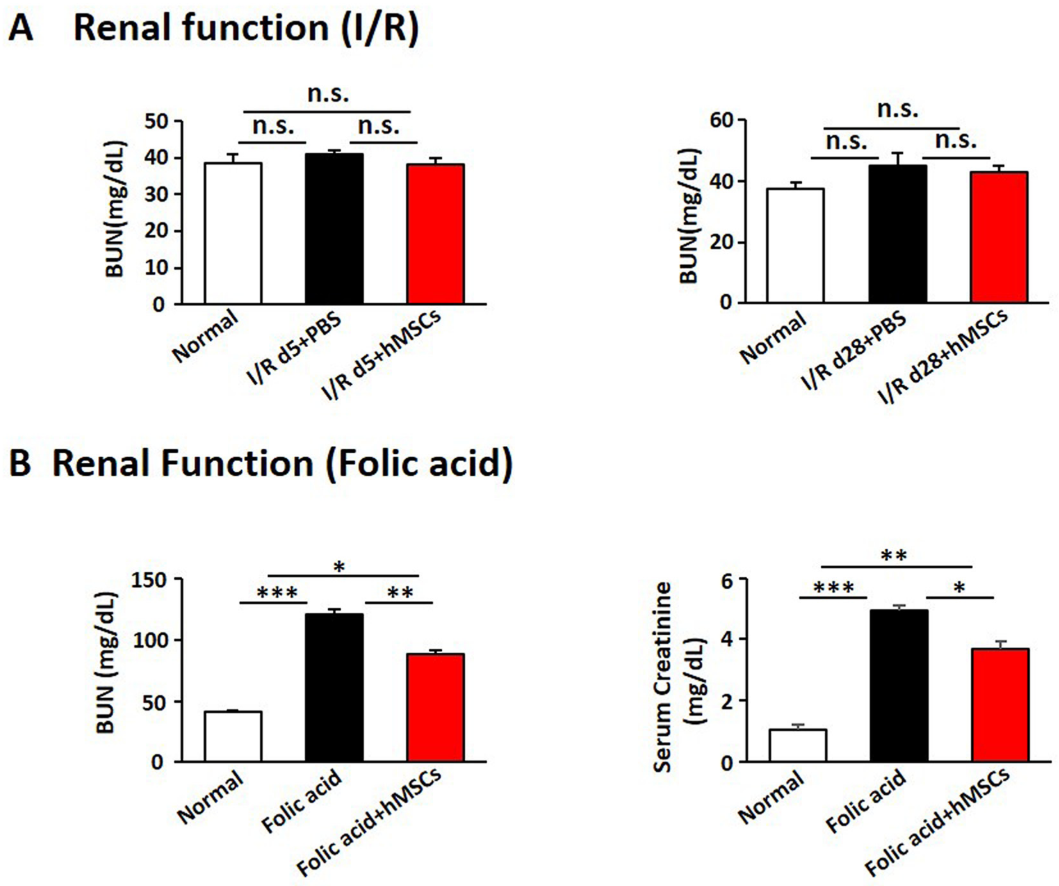

Supplementary Figure 2: Changes of renal function. **(A)** Change of serum urea nitrogen with or without hAD-MSC treatment on day5 (left) and day 28 (right). **(B)** Change of serum urea nitrogen (left) and creatinine (right) in AKI induced by folic acid. N=5/group. Values were means ±SEM. *$P < 0.05$, **$P < 0.01$, ***$P<0.001$.
